# Transition from open partial nephrectomy directly to robotic surgery: experience of a single surgeon to achieve “TRIFECTA”

**DOI:** 10.1590/S1677-5538.IBJU.2019.0101

**Published:** 2020-07-31

**Authors:** Tiago Mendonça Lopez Castilho, Gustavo Caserta Lemos, Jonathan Doyun Cha, José Roberto Colombo, Oliver Rojas Claros, Maria Beatriz Lemos, Arie Carneiro

**Affiliations:** 1 Hospital Israelita Albert Einstein São Paulo SP Brasil Hospital Israelita Albert Einstein, São Paulo, SP, Brasil

**Keywords:** Kidney Neoplasms, Carcinoma, Renal Cell, Robotics, Nephrectomy

## Abstract

**Introduction:**

Recent data suggest that robotic platform has become the most accessible minimal invasive surgery even for surgeons without previous training in laparoscopy. Laparoscopic partial nephrectomy (LPN) is a well-stablished procedure, however, with high level of complexity and long learning curve that limit its use.

**Objective:**

To describe safety, efficiency and learning curve of a single surgeon without previous experience in LPN to reach “TRIFECTA” at robot-assisted partial nephrectomy (RAPN).

**Patients and Methods:**

This is a retrospective study, with prospective data collection of 101 patients submitted to RAPN by a single surgeon. In order to analyze the learning curve, sample was chronologically divided in two phases: first phase: P1: 50 first patients, second phase: P2: 51 subsequent patients. TRIFECTA was defined as: ischemia time lower than 25 minutes, negative surgical margin and absence of severe complications (Clavien >2).

**Results:**

Mean age of patients was 54 years (SD=11.85), median tumor size was 32mm (SD=17) and surgery was performed with zero ischemia time in 33.6% of patients (29.8% at P1 and 40.9% at P2). Demographic data of patients were similar between both groups, except tumor size (P1=27.5mm vs. P2=35.3mm; p=0.02) and body mass index (BMI) (P1=26.6kg/m^2^ vs. P2=29kg/m^2^; p=0.03). Rate of bleeding, surgical time, presence of positive margin and peri-operatory surgical complications were similar in both phases. TRIFECTA was higher in P2 in relation to P1 (P1: 58% vs. P2: 87.8%; p=0.002) and median time of hot ischemia was significantly lower at P2 (P1: 17.3 vs. P2: 11.7; p=0.02). At multivariate analysis independent factors related to TRIFECTA included: chronological phase (OR 10.74; 95% IC: 1.63-70.53; p=0.013) and tumor size (OR 0.95; 95% IC: 0.91-0.99; p=0.024).

**Conclusion:**

RAPN seems to be safe and efficient with good functional and oncological results (TRIFECTA) since the beginning. Experience improvement was related to treatment of larger tumors, higher proportion of patients with zero ischemia and higher rate of TRIFECTA.

## INTRODUCTION

Gold standard treatment of localized renal carcinoma is surgery, with oncologic and functional benefits very well stablished and validated (1, 2). Nephron-sparing surgery (NSS) is prefered and must always be performed whenever possible in the treatment of renal tumors (2). Most adequate access depends on the characteristics of the tumor and surgeon’s experience (3).

Minimally invasive techniques have been more used in order to benefit patients with lower time of recovery, less pain and lower rate of complications related to surgical wound (4).

Many surgeons with basic training in laparoscopy are able to perform an ablative surgery such as radical nephrectomy with safety and good results. However, partial nephrectomy is a procedure with high complexity where it is mandatory to remove the tumor and to reconstruct renal parenchyma with hemostasis in a very short time interval. It is necessary the presence of a high trained team (3). The concept of TRIFECTA (negative surgical margins, ischemia time lower than 25 minutes and absence of severe complications) was described by Gill et al. and it is used to evaluate the surgical success of LPN (5, 6).

Transition from LPN to RAPN has been well studied (7). However, direct use of RAPN by surgeons without previous experience in LPN has not been well described in literature.

## OBJECTIVE

To describe the safety, efficiency and learning curve of a single surgeon without previous experience in LPN of the first 100 cases treated by robot-assisted partial nephrectomy (RAPN).

Primary objective: to evaluate complication rate, time of hot ischemia (THI) and TRIFECTA rate.

Secondary objectives: to evaluate tumor size, complexity (nephrometry), surgical time, bleeding and rate of transfusion.

## PATIENTS AND METHODS

### Design of study, local, ethics

This is a retrospective study of data collected prospectively with a single arm of patients submitted to robot-assisted partial nephrectomy from January 2009 to June 2016, in a private hospital in Brazil by a single surgeon (GCL) without previous training in video-laparoscopy. The study was approved by the institutional ethics committee (76547917.5.0000.0071).

The data of the present study are presented in a descriptive form. In order to analyze the learning curve patients were divided chronologically in two groups, that were compared in all aspects. Sample was chronologically divided in two phases (first phase - P1=50 patients; second phase - P2=subsequent 51 patients).

### Surgical technique

The procedure was standardized and performed according to previous study (3). Initially, all patients with high and intermediate complexity (RENAL score >6) were performed with clamping of renal artery. After the first 30 cases, well defined non-hilar nodules, with endophytic component of up to one centimeter were treated ac-cording to this standardization: renal hilum dissection isolating the renal artery and enucleation with clamping only if necessary.

Reconstruction of renal parenchyma was performed with two layers (medullary and cortical) with continuous suture (initially with Vycril® and posteriorly with V-loc suture). Early de-clamping (following medullary suture) was routinely performed after patient # 72.

### Analyzed variables and outcomes

Abdominal computer tomography was reanalyzed by two urologists (TC, PPK) and the nodules were classified according to nephrometry R.E.N.A.L. score (8), and were considered with high complexity when score >6.

During the pre-operatory period, the following information were obtained: age, sex, body mass index (BMI), race, laterality, comorbidities, ASA (physicil status of the American Society of Anesthesiologists), renal function and tumor size. The tumors were classified as endophytic when more than 50% of its volume was located inside the renal parenchyma.

During surgery, the following data were collected: time of use of robot, bleeding (mL), complications, time of hot ischemia (THI), need of clamping, early de-clamping.

Following surgery, it was collected data related to serum hemoglobin (Hb), hematocrit (Ht), renal function and complications.

Complications were recorded and classified according to Clavien-Dindo system, and the need of conversion to open surgery was classified as an adverse event (8).

Renal function of patients were evaluated by serum creatinine and creatinine clearance, calculated by the Cockroft-Gault formula. Evolution (or loss of renal function) was evaluated by an analysis in three moments: before surgery, recent post-operatory period (one to three months after surgery) and the last data during follow-up. Reduction of renal function was considered when creatinine clearance was lower than 80% of initial.

Surgical success (TRIFECTA) was defined, according to Gill et al., as: ischemia time lower than 25 minutes, negative surgical margin and absence of severe complications (Clavien>2) (4). If the patient did not meet all those criteria it was considered as unsuccessful.

### Statistical Analysis

All statistical analysis were performed using the SPSS software for Windows version 20.0 (SPSS, Chicago, IL, USA). Significance level was <0.05.

Numeric variables were expressed as median and standard deviation. Non-parametric numeric variables were submitted to Mann-Whitney U test. In order to evaluate renal function in the three moments (related samples) it was used the Friedman paired test. For categorical variables, it was used the Chi-square test or exact of Fisher, depending on the quantity of positive at outcome (when expected values in contingence table cells were lower than 5). Multivariate analysis with binary logistic regression was performed to evaluate the factors related to TRIFECTA.

## RESULTS

Median age of patients was 54 years (SD=11.85); median tumor size was 32mm (SD=17). Demographic characteristics of patients were similar between both phases except for the tumor size (P1:27.5mm vs. P2: 35.3mm; p=0.02) and body mass index (BMI) (P 1:26.6 kg/m^2^ vs. P2:29 kg/m^2^; p=0.003) (Table-1).

**Table 1:** Demographic data.

	Phase 1		Phase 2		
		N		N	P
Age, median (SD)	55.4 (10.4)	50	53.8 (13.1)	51	0.49&
Male, %	80.00%	40	72.50%	37	0.37#
Female, %	20.00%	10	27.50%	14	
Bmi, median (SD)	26.6 (2.8)	42	29 (3.4)	28	0.003&
Laterality					0.51#
Right, %	38.30%	18	44.90%	22	
Left, %	61.70%	29	55.10%	27	
Renal score					0.62#
Low renal score, %	70.3%	26	60%	24	0.34#
Moderate renal score moderate, %	21.6%	8	27.5%	11	0.55#
High renal score, %	8.1%	3	12.5%	5	0.71$
Size, median (SD)	27.5 (12.6)	47	35.3 (19.5)	44	0.02&
ENDOPHITIC (>50% intraparenchymal), %	79.10%	41	93.2%	34	0.056#
Pre cr, medium (sd)	1.0 (0.3)	22	0.86 (0.2)	31	0.08&
Pre-op clearence, medium (SD)	103.7 (38.3)	19	120.2 (35)	27	0.13&
ASA 1 Classification	34.1%	15	32.7%	16	0.453#
ASA 2 Classification	63.6%	28	59.2%	29	
ASA 3 Classification	2.3%	1	8.2%	4	

# = Chi-square test;

& = Student t test;

$ = Fisher exact test

Tumor complexity was similar between groups. However, in a sensitive analysis according to complexity level, it was observed that at P1 there was a higher proportion of patients with low complexity (70.3% vs. 60%, p=0.34) and a lower proportion of high complexity (8.1% vs. 12.5%, p=0.71); 79.1% at P1 were endophytic (more than 50% of tumor volume intraparenchymal) and 93.2% at P2 (p=0.056) (Table-1).

In a paired analysis of both phases it was not observed any difference of creatinine clearance before and after surgery (P1: 108.9 vs. P2: 109mL/min; p=0.987 and P1:125.2 vs. P2 126.8; p=0.775 (Table-2).

**Table 2:** Paired analysis of clearance of creatinine.

	PHASE 1			PHASE 2		
		N	p		N	p
PRE-OP CLEARENCE PRE-OP, MEDIUM (SD)	109 (40.7)	16	0.987&	126.8 (37.4)	19	0.775&
POST-OP CLEARENCE, MEDIUM (SD)	108.9 (45.7)	16		125.2 (36.8)	19	

& = Paired t Student test

Mean ischemia time was significantly lower at P2 (P1: 17.3 vs. P2: 11.7 minutes; p=0.02) and the proportion of surgery with zero ischemia was proportionally lower at P1 (29.8% at P1 vs. 40.9% at P; p=0.259).

Bleeding, surgical time, presence of positive margin and peri-operatory complications were similar in both phases (Table-3).

**Table 3:** Peri-operatory data.

	PHASE 1		PHASE 2		
		N		N	P
Surgical time, medium (SD)	114.3 (29.7)	44	120 (48.5)	31	0.46&
Clamping time , medium (SD)	17.3 (13.1)	46	11.7 (10.9)	49	0.02&
Use of clamp					0.259#
With clamp	70.2%	33	58.1%	29	
Without clamp	29.8%	14	40.9%	20	
TRIFECTA 25 min. %	58%	29	87.8%	36	0.002#
Bleeding, medium (sd)	295.6 (372)	39	375.3 (282)	38	0.88&
Positive margin, %	2.3%	1	0	0	0.344#
Clavien 1/2, complication, %	16%	8	7.8%	4	0.506#
Clavien 3/4, complication %	2%	1	0	0	

# \Chi-square test;

& Student t test

Clavien 1 and 2 complications occurred in 16% of Phase 1 patients and in 7.8% of Phase 2 patients (p=0.506). Only one patient presented a severe complication at P1 (Clavien 3 or 4). This patient presented hematoma at the renal site that was treated with percutaneous drainage. One patient at P1 needed conversion to open surgery due to difficulty to expose the nodule (it was located at the posterior region of the upper pole of right kidney). Two patients showed positive margins (one identified at the intra-operatory freezing biopsy and the other with final diagnosis of oncocytoma where the margin was only identified at the pos-operatory anatomopathological exam). These patients are been followed, and showed no recurrence for 20 months. At P2 it was not observed any patient with severe complication or positive margin (Table-3).

Treatment success (TRIFECTA) was significantly higher at P2 in relation to P1 (P1: 58% vs. P2: 87.8%; p=0.002) (Table-3).

In the multivariate analysis, the independent factors related to TRIFECTA included: phase and tumor size (Table-4). Other analyzed aspects showed no correlation (BMI, pre-operatory creatinine, nephrometry and endophytic/ exophytic localization).

**Table 4:** Multivariate logistic regression (outcome: to reach 25 min. Trifecta).

	OR (CI 95%)	P
PHASES	10.74 (1.63-70.53)	0.013
Size	0.95 (0.91-0.99)	0.024
BMI	0.94 (0.76-1.15)	0.572
Endo/Exophytic	0.07 (0.00-1.37)	0.080

## DISCUSSION

This study shows the experience and evolution of a surgeon without previous experience in video-laparoscopy surgery, during the 100 first cases of RAPN. Since the beginning, the results were satisfactory, with significant improvement of TRIFECTA rate at the second phase (after 50 cases), especially due to the reduction of ischemia time. With experience gain, larger tumors were operated and the no-clamping technique was more used.

The steep learning curve, of the transition from open partial nephrectomy to laparoscopy has become one of the most limiting factor for the use of minimally invasive techniques for this procedure, used only in a few centers with a big number of surgeries and specialized team. Robotic platform is proving to be an important tool to change paradigms, mainly in urologic surgery and has made minimally invasive surgery more available, even for surgeons without previous experience in video-laparoscopy (9, 10). This fact is related to the characteristics of this technology, that allows for a shortening of the learning curve, assuring the same functional and oncological results of conventional surgery (5, 11, 12).

With the advances of minimally invasive surgery, it is possible to reduce the hospitalization time and costs, not only of the surgery itself but the social costs, as described by the series of Chang et al. they demonstrated a medium withdrawal time of 35 days from work, from 7 to 92 days, with a medium salary loss of US$ 10.152 in the United States (13).

In the last years, a great number of institutions acquired the robotic platform, even in developing countries. Radical prostatectomy is the main surgery in most programs, and high volume surgeons, used to conventional surgery, are using it more frequently. The transition from radical prostatectomy to robot-assisted was widely studied and the data showed that it is not necessary previous experience with laparoscopic radical prostatectomy (14).

Ghani et al. reported an expressive increase of the use of robotic platform instead of laparoscopic surgery in partial nephrectomy in the US. It was also suggested that it is possible to move from open surgery to robot-assisted without learning video-laparoscopy (9) and the number of urologists that are using minimally invasive technique is increasing. This fact was also observed in our institution where the robotic platform allowed for a wider access to minimally invasive procedure.

The promising results of our study since the first patients may be explained by the adoption of a structured program with PROCTOR to follow the procedures until consolidation of learning of surgeons, allowing for a safe and efficient transition from conventional technique to robot-assisted.

Many surgeons, especially those from developing countries, have limited training in laparoscopy and use open conventional surgery routinely, particularly in more complex cases. With the dissemination of the robotic platform and wider availability, more and more surgeons without previous training in laparoscopy are been trained to use the robotic platform (15).

The rate of conversion to open surgery, complications and positive margins are closely related to the experience of the surgeon. Similar to our study, Khalifeh et al. also reported conversion from open surgery at first phase, with absence of any type of complications, positive margins and ischemia time longer than 25 minutes, for partial nephrectomy (16). Similarly, Haber et al. (12) reported all conversions at the first 20 patients.

Hot ischemia time has already been widely studied and it was stablished that the lower the time the better the functional preservation (17). However, the ideal and safe limit is still being debated. Originally, the time of 30 minutes was considered the limit time for preservation of the renal parenchyma (18), but this value has been reduced along time. The most used concept in several studies and in our series was proposed by Gill et al. that stablished a goal of time lower than 25 minutes (4). Medium time of hot ischemia in our series was 22 minutes. We observed a significant reduction of ischemia time (p=0.034) when we compared P1 and P2 patients; however, since the first cases, we have observed “ideal” times compared to international series (19). Gill et al. reported a lowering of the medium time of hot ischemia from 32 to 14 minutes only after 500 patients operated (4). Our initial results probably were superior than those described by Gill due to the model of implantation of the program. Our program had a PROCTOR in all procedures to ensure standardization, quality and safety since the first cases.

In our series, at P2 there was an important change of ischemia results, that certainly was reflected in the TRIFECTA results. This occurred mainly due to change of approach and more frequent use of no-clamping technique and early de-clamping, that certainly was the main factor related to the increase of TRIFECTA at Phase 2. Different from others authors, we observed a significant reduction of the ischemia time due to a wider use of the no-clamping technique and not due to lower time of suture (17): more important than agility, experience made us confident to change our approach and to perform a high quality surgery even with more bleeding.

At P2 we observed a higher rate of bleeding and this may also be justified by the increased use of the no-clamping technique. However, this difference had no clinical significance and did not alter the rate of transfusion.

Rate of complications at literature varied from 8% to 22% (20, 21) and in our was 5.88% with no Clavien 3 or 4 at P2.

In relation to oncological results, we observed positive margins in two patients, exclusively at P1. According to good practices of oncologic surgery, it is essential to assure negative margins. Otherwise, at literature it was not possible to demonstrate an increased risk of local recurrence or progression to metastatic disease in patients submitted to partial nephrectomy with positive margins (22). Recent studies investigated the impact of the presence of microscopic positive surgical margins that suggested that their presence was not related necessarily to residual disease (23, 24). Therefore, according to current scientific knowledge, the presence of positive surgical margin at post-operatory of partial nephrectomy is still being highly debated, and should not be used as the only or the main indicator of efficiency of oncologic surgery (25).

Differently of other series, such as Carneiro et al., that demonstrated a significant increase of renal complexity and size of tumor, as well as endophytic cases in later groups (7), in our study we have not identified any difference of nephrometry between the two phases, except by the tumor size, where it was observed an increase at P2 as well as more endophytic cases.

At Gill et al. series, evaluation of progression of renal function in three different moments in the same patient showed in the last patients submitted to laparoscopic partial nephrectomies a lowering of medium glomerular filtration rate (GFR) of 11% (4). In our series, there were no significant alterations of GFR regardless the moment, demonstrating the safety of functional preservation (Table-1).

Similar to the study of Khalifeh et al., TRIFECTA rate above 60% was reached in special after the first 50 patients, similar to other studies, that reported a short and safe learning curve (16, 26). We considered our results favorable to acquisition of a learning curve without compromising the final results; however, we agree that it is necessary more 30 or 40 cases to reach stable and consistent results and mastering the technique (16, 26). And as quoted by Larcher et al., after 150 patients it is not observed any additional improvement of ischemia time (27).

At multivariate analysis, the independent factors of TRIFECTA included the experience of the surgeon and the size of the tumor. Therefore, we suggest that in the beginning of the learning curve tumors with lower volume and lower nephrometry must be selected, although in the last aspect in our series that fact was not observed.

It is important to highlight that this study has some limitations. This is a small series with a short follow-up period compared to some already published in big centers of developed countries.

## CONCLUSIONS

This study demonstrated that the transition from open nephrectomy to robot-assisted without learning laparoscopic nephrectomy is safe, with satisfactory functional and oncologic results since the beginning (TRIFECTA). Increased experience lead to a higher proportion of patients operated with zero ischemia and TRIFECTA.

## Abbreviations

LPN: laparoscopic partial nephrectomy
RAPN: robot-assisted partial nephrectomy
BMI: body mass index
NSS: nephron sparing surgery
IT: ischemia time
HIT: hot ischemia time
Hb: Serum Hemoglobin
Ht: Hematocrit
GFR: Glomerular filtration rate
ASA: Classification of physical status of the “American Society of Anesthesiologists”
